# Supplementary material for: Sigma-2 Receptor Ligand Binding Modulates Association between TSPO and TMEM97
Source: Int J Mol Sci. 2023 Mar 28;24(7):6381. doi: 10.3390/ijms24076381 (PMC10093951; doi:10.3390/ijms24076381)
Supplement: Supplementary file 1 [file ijms-24-06381-s001.zip › ijms-2272181_Final-supplementary V.2.pdf]

**Supplementary Information**

# Sigma-2 Receptor Ligand Binding Modulates Association between TSPO and TMEM97

Bashar M. Thejer [1,2,†], Vittoria Infantino [3,†], Anna Santarsiero [3], Ilaria Pappalardo [3], Francesca S. Abatematteo [4], Sarah Teakel [1], Ashleigh Van Oosterum [5,6], Robert H. Mach [7], Nunzio Denora [4], Byung Chul Lee [8,9],Nicoletta Resta [10], Rosanna Bagnulo [10], Mauro Niso [4], Marialessandra Contino [4], Bianca Montsch [11],Petra Heffeter [11], Carmen Abate [4,12,*] and Michael A. Cahill [1,13]

## Affiliations

1. School of Dentistry and Medical Sciences, Charles Sturt University, Wagga Wagga, NSW 2678, Australia
2. Research and Development Department, The Ministry of Higher Education and Scientific Research,
   Baghdad 10065, Iraq
3. Department of Science, University of Basilicata, Viale dell'Ateneo lucano 10, 85100 Potenza, Italy
4. Department of Pharmacy-Drug Sciences, University of Bari 'ALDO MORO', Via Orabona 4, 70125 Bari, Italy
5. Life Sciences and Health, Faculty of Science, Charles Sturt University, Wagga Wagga, NSW, 2650, Australia
6. School of Medicine and Psychology, Australian National University, Florey Building, 54 Mills Road, Acton, ACT 2601, Australia
7. Department of Radiology, Perelman School of Medicine, University of Pennsylvania, Philadelphia, PA 19104, USA
8. Department of Nuclear Medicine, Seoul National University Bundang Hospital, Seoul National University College of Medicine, Seongnam 13620, Republic of Korea
9. Center for Nanomolecular Imaging and Innovative Drug Development, Advanced Institutes of Convergence Technology, Suwon 16229, Republic of Korea
10. Dipartimento di Medicina di Precisione e Rigenerativa e Area Jonica (DIMePRe-J), Università degli Studi di Bari 'ALDO MORO', Piazza Giulio Cesare, 70124 Bari, Italy
11. Center for Cancer Research and Comprehensive Cancer Center, Medical University of Vienna, Borschkegasse 8a, 1090 Vienna, Austria
12. Consiglio Nazionale delle Ricerche (CNR), Istituto di Cristallografia, Via Amendola, 70125 Bari, Italy
13. ACRF Department of Cancer Biology and Therapeutics, The John Curtin School of Medical Research, The Australian National University, Canberra, ACT 2601, Australia

* Correspondence: carmen.abate@uniba.it

† These authors contributed equally to this work.

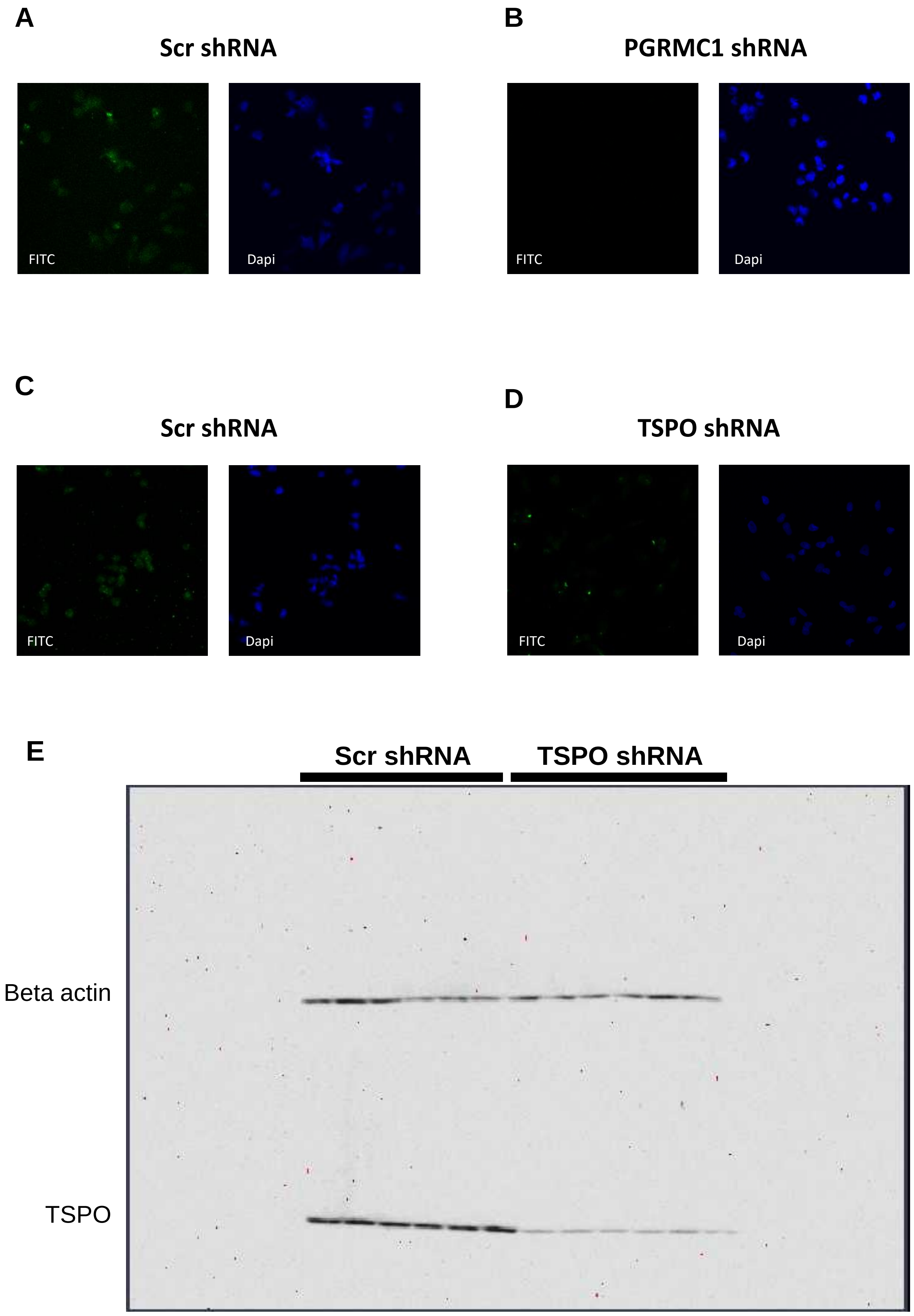


**Figure S1. PGRMC1 and TSPO knockout by shRNA.**

(**A**) Represents immunohistochemistry (IHC) analysis of PGRMC1 expression in Scr shRNA MP cell lines. Images were captured by confocal microscopy using primary PGRMC1 antibody at 1:100 concentration and

FITC-conjugated secondary antibody (Sigma, F8521). Blue represents DAPI staining of DNA. Each discrete blue patch represents one cell nucleus, imaged at 20x magnification.

(**B**) Represents IHC analysis after attenuation the level of PGRMC1 by shRNA using similar antibodies, incubation conditions and confocal microscopy setting. Scale follows (**A**).

(**C**) Illustrates the level of TSPO in MP cell lines using goat anti-TSPO primary antibody (Invitro Technologies, NOVNB10041398) at 1:100 and FITC anti-goat secondary antibody. Scale follows (**A**).

(**D**) A negative control that shows the attenuation of TSPO by shRNA. Again, similar conditions were used for signal detection.

(**E**) Image of the original Western blot that was quantified to generate Figure 1C.

<table>
<tr><th>TSPO</th><th>S2R</th></tr>
<tr><td>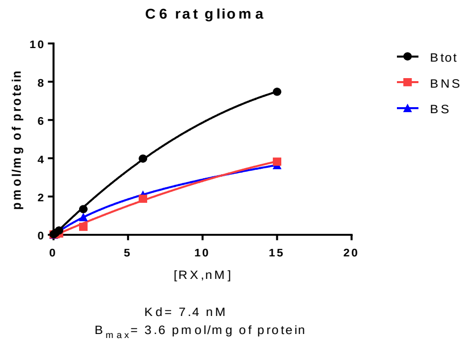
</td><td>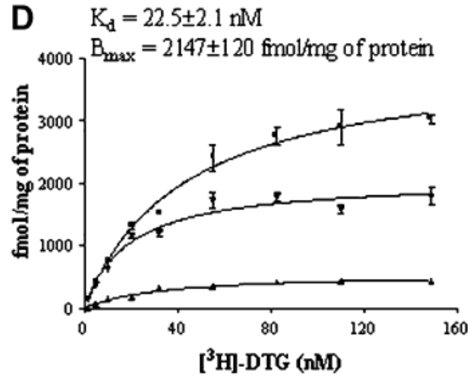
</td></tr>
<tr><td>A</td><td>B</td></tr>
<tr><td>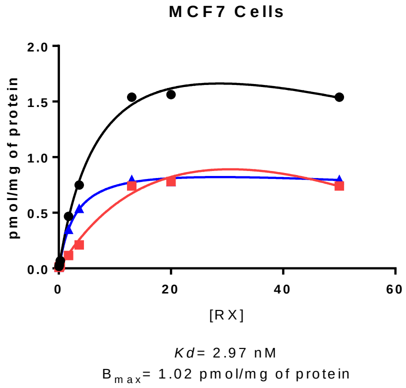
</td><td>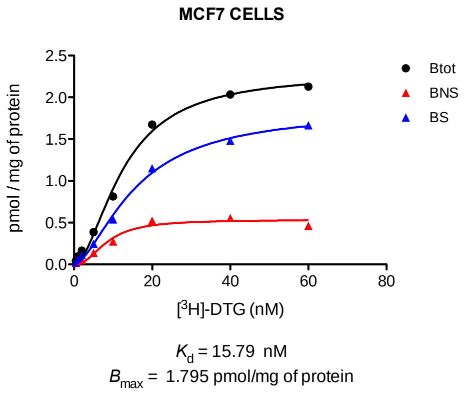
</td></tr>
<tr><td>C</td><td>D</td></tr>
</table>

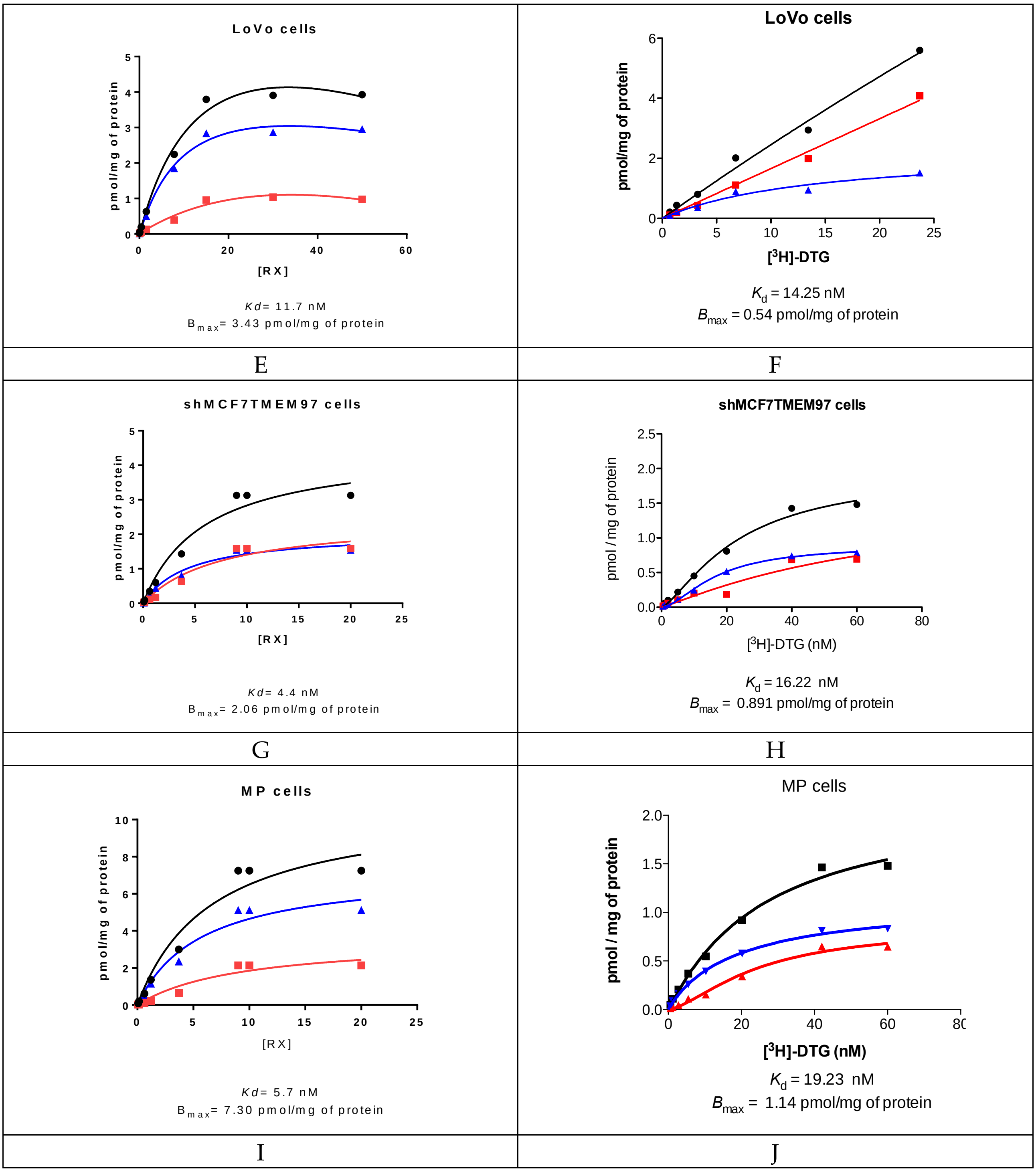


**Figure S2.** Saturation analysis with the appropriate radioligand of TSPO and S2R in membrane preparations from rat C6 (**A** and **B**), MCF7 (**C** and **D**), LoVo (**E** and **F**), shMCF7TMEM97 (**G** and **H**) and MP (Mia PaCa-2) (**I** and **J**) cell lines.

(**A**, **C**, **E**, **G** and **I**) For TSPO saturation binding assays [$^3$H]PK1195 has been used as radioligand and 10 µM PK11195 to measure the no-specific binding;

(**B**, **D**, **F**, **H** and **J**) For S2R saturation binding assays [$^3$H]DTG has been used as radioligand and 10 µM DTG to measure the no-specific binding.

The dissociation constant $K_d$ and the receptor density (ligand binding sites) $B_{max}$ were measured by the nonlinear fitting of the specific binding vs RX concentration using Prism software.

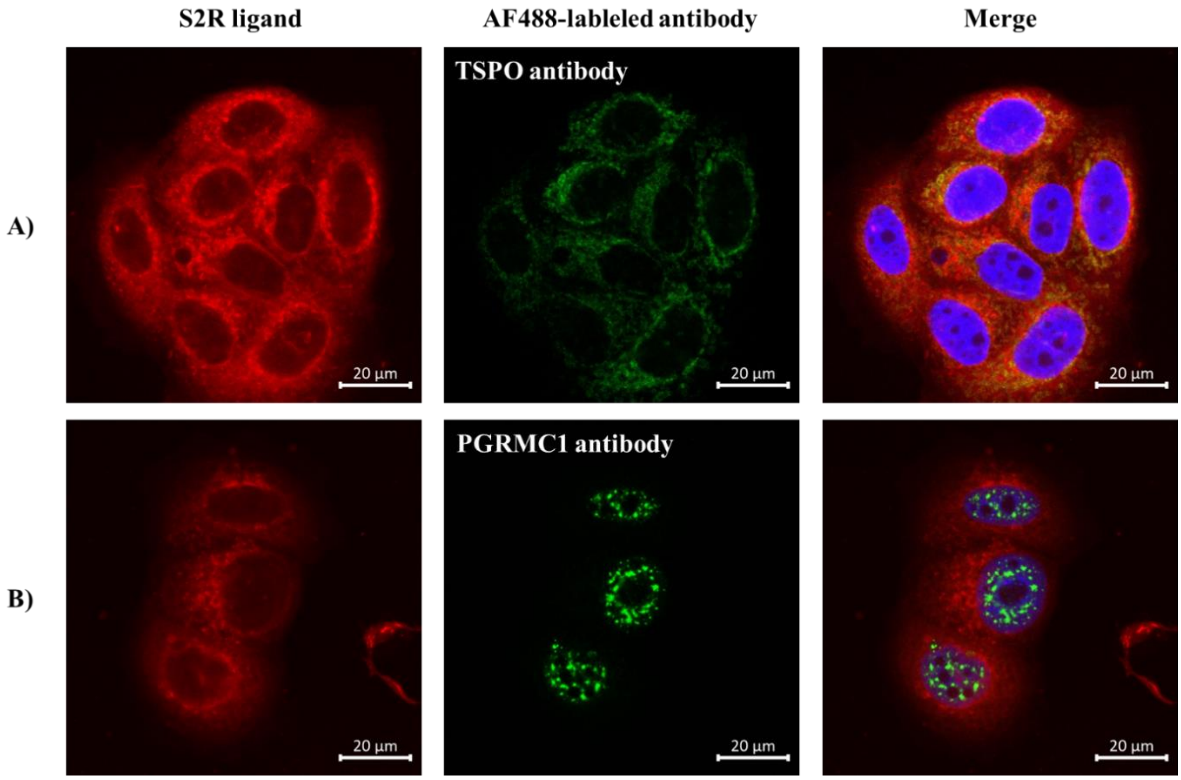


**Figure S3**. Representative confocal microscopy images show the binding site of the CY5-labeled S2R ligand, co-stained with (**A**) TSPO- as well as (**B**) PGRMC1-specific antibodies in MCF7 cells. Cells were pre-incubated with 10 µM of the selective S1R agonist (+)-pentazocine for 2 h to mask residual S1R expression. This was followed by 1 h incubation with 5 µM for the S2R ligand (shown in red). Then cells were fixed with paraformaldehyde, stained for TSPO or PGRMC1 expression (shown in green) as well as nuclei (by DAPI, shown in blue) and analyzed by confocal microscopy (scale bar: 20 µm).

**Table S1. STR profiling of MCF7 and MP cell lines.** STR typing of DNA obtained from 2 samples collected was performed with the NGM amplification kit (TFS; see Materials and methods). The effectiveness of autosomal STR typing, expressed as the number of successfully typed loci, is shown in the table above. The STRs characterized in ATCC are bold.

| Locus designation NGM Select kit | Chromosome location | Allelels MIACAPA-2 | Allelels MCF7 |
|---|---|---|---|
| D10S1248 | 10q26.3 | 14/15 | 14/14 |
| **vWA** | **12p13.31** | **15/15** | **14/15** |
| **D16S539** | **16q24.1** | **10/13** | **11/12** |
| **D2S1338** | **2q35** | **25/25** | **21/23** |
| **Amelogenin** | **X:p22.1-22.3 Y:p11.2** | **XX** | **XX** |
| D8S1179 | 8q24.13 | 16/19 | 10/14 |
| D21S11 | 21q11.2-q21 | 29/31.2 | 30/30 |
| D18S51 | 18q21.33 | 12/12 | 14/14 |
| D22S1045 | 22q12.3 | 16/16 | 15/16 |
| D19S433 | 19q12 | 15/15 | 13/14 |
| **TH01** | **11p15.5** | **9/10** | **6/6** |
| FGA | 4q28 | 22/22 | 23/25 |
| D2S441 | 2p14 | 14/15 | 10/14 |
| D3S1358 | 3p21.31 | 16/16 | 16/16 |
| D1S1656 | 1q42.2 | 15/17.3 | 11/15.3 |
| D12S391 | 12p13.2 | 19/19 | 18/20 |
| SE33 | 6 | 16/16 | 16/18 |
